# A Manual of Fevers

**Published:** 1911-12

**Authors:** 


**A Manual of Fevers.** By Claude Buchanan Ker, M.D. Pp. xiii, 314. London: Oxford Medical Publications. 1911.

This manual, written by an expert on infectious diseases as they are seen in Edinburgh, is intended for the use of students who propose to take out the statutory course of fevers at an isolation hospital. It cannot fail also to be of use to its owner when afterwards engaged in the work of general practice. The diseases to be treated are due to living organisms, and are approached from this point of view. Several of them are known to be bacteria, some are probably protozoa, some have yet to be discovered. The elements of infection and immunity are described, and fever is defined as " a response in metabolism to the invasion of micro-organisms and a toxic disturbance of the regulation of temperature." Now that the phenomena of infection are so well understood, we think that the useless and mysterious word " Fomites " might well drop out of the English language. The author should be encouraged in his endeavour to belittle the disease rubella, of which the infection is short-lived, and is destroyed by ordinary domestic cleaning. He holds that although we are occasionally confronted with rashes which defy classification, there is no sufficient ground for the establishment of the " fourth disease " of Clement Dukes.

